# Community perceptions of vaccine advocacy for children under five in rural Guatemala

**DOI:** 10.1371/journal.pgph.0000728

**Published:** 2023-05-22

**Authors:** Joshua T. B. Williams, Kelsey Robinson, Elizabeth Abbott, Neudy Rojop, Michelle Shiffman, John D. Rice, Sean T. O’Leary, Edwin J. Asturias

**Affiliations:** 1 Center for Health Systems Research, Denver Health, Denver, Colorado, United States of America; 2 Department of Pediatrics, University of Colorado Anschutz Medical Campus, Aurora, Colorado, United States of America; 3 Colorado School of Public Health, Aurora, Colorado, United States of America; 4 Immunize Colorado, Aurora, Colorado, United States of America; 5 Fundacion para la Salud Integral de los Guatemaltecos, Coatepeque, Coatepeque, Guatemala; 6 Adult and Child Consortium for Health Outcomes Research and Delivery Science (ACCORDS), Aurora, Colorado, United States of America; 7 Section of Pediatric Infectious Diseases, Department of Pediatrics, Children’s Hospital Colorado, Aurora, Colorado, United States of America; Stellenbosch University, SOUTH AFRICA

## Abstract

Historically, partnerships with community leaders (e.g., religious leaders, teachers) have been critical to building vaccination confidence, but leaders may be increasingly vaccine hesitant. In rural Guatemala, the extent of vaccine hesitancy among community leaders is unclear, as are their perceptions of advocacy for childhood vaccines. We sought to: (i) compare Guatemalan religious leaders’ and community leaders’ attitudes toward childhood vaccines, (ii) describe leaders’ experiences and comfort with vaccination advocacy, and (iii) describe community members’ trust in them as vaccination advocates. In 2019, we surveyed religious leaders, other community leaders, and parents of children under five in rural Guatemala. We recorded participant demographic information and assessed participant vaccine hesitancy regarding childhood vaccines. We analyzed data descriptively and via adjusted regression modeling. Our sample included 50 religious leaders, 50 community leaders, and 150 community members (response rate: 99%); 14% of religious leaders and community leaders were vaccine hesitant, similar to community members (P = 0.71). In the prior year, 47% of leaders had spoken about vaccines in their formal role; 85% felt responsible to do so. Only 28% of parents trusted politicians “a lot” for vaccine advice, versus doctors (72%; P < 0.01), nurses (62%; P < 0.01), religious leaders (49%; P < 0.01), and teachers (48%; P < 0.01). In this study, religious leaders and community leaders were willing but incompletely engaged vaccination advocates. Most community members trusted doctors and nurses a lot for vaccination advice; half trusted teachers and religious leaders similarly. Public health officials in rural Guatemala can complement efforts by doctors and nurses through partnerships with teachers and religious leaders to increase vaccination confidence and delivery.

## Introduction

Immunization is crucial to child health in developing countries, preventing 2–3 million deaths per year [1]. Despite progress in international vaccination coverage over the past decades, infant vaccination rates have stalled around 85–86% [2]. In Guatemala, only 59% of children aged 12–23 months were fully immunized according to recent national surveys [3]. Multiple barriers to timely vaccination exist in global health contexts, and vaccine hesitancy is a growing public health threat [1, 3]. Previously, we validated a vaccine hesitancy tool among Guatemalan parents of children under five, finding one in six parents were hesitant and children of hesitant parents had an increased adjusted odds (aOR 2.5; 95% CI: 1.2, 5.4) of under-vaccination at age 19 months, referent to children of non-hesitant parents [4]. Other investigators have noted similar concerns. In a multi-country survey of parents of children under five conducted from 2016–2018, 72% of Guatemalan respondents agreed or strongly agreed that they were concerned about vaccine side effects [5]. A 2016 survey of rural and urban Guatemalan parents of children 6 weeks to 6 months old found generally positive attitudes toward infant vaccines, but 59% thought other parents did not have their children up to date with recommended vaccines, and 35% agreed or were unsure whether newer vaccines carry more risks than older vaccines [6]. Religion may also contribute to parental vaccine hesitancy; a global survey of adults, including samples from Central and Latin America, found over 10% of individuals disagreed that vaccines were compatible with their religious beliefs [7].

Multiple tactics exist for countering vaccine hesitancy in developing countries [8]. Yet, community engagement with civic leaders and religious leaders has been paramount for decades [8]. The eradication of smallpox is attributed first and foremost to the work of community leaders and members who became lay health advocates, educators, and vaccinators [9]. Engagement has increased uptake of other novel vaccines, such as those for Meningitis A, Yellow Fever, and Ebola, in South American and African low- and middle-income countries [10]. Engagement continues to be equally critical for childhood polio eradication efforts, especially in conflict-prone areas of Nigeria, Pakistan, and Afghanistan [11–14]. One randomized controlled trial of community-engaged support versus standard of care in Pakistan found a 10% difference in oral polio vaccination rates between children randomized to community mobilization and those assigned to usual care [15].

Despite a history of successful community-engaged vaccination campaigns across the world, community leaders’ perceptions of child vaccines may not be uniformly positive. In the aforementioned 2016 study of Guatemalan parents of children 6 weeks to 6 months old, 32% of respondents thought community leaders (e.g., religious or political leaders, teachers) did not support vaccinations for infants and children [6]. However, the study did not formally assess attitudes among leaders themselves or further explore parent preferences for vaccination advocates. Thus, we conducted a cross-sectional survey to: (i) compare rural Guatemalan community members’, community leaders’, and religious leaders’ attitudes toward childhood vaccines, (ii) describe community leaders’ and religious leaders’ experiences and comfort with vaccine advocacy, and (iii) describe community members’ perceptions of trust in community leaders and religious leaders as vaccine advocates.

## Materials and methods

### Study setting

The study occurred in the coastal lowlands of southwestern Guatemala near the border with Chiapas, Mexico. This region is named the Southwest Trifinio (triangle) for the confluence of three departments (i.e., states) of San Marcos, Quetzaltenango, and Retalhuleu; about 30,000 people live in this area and primarily work in agribusiness enterprises to cultivate crops for export [16]. In 2011, a rapid anthropologic assessment procedure based on over 300 structured interviews with family members and community leaders identified potable water, health care services, and education as three priorities for the community [16]. Locations and contact information for churches, schools, and major employers were also noted at this time [16].

### Study design and survey sample

We performed a cross-sectional pilot study of community members (n = 150), religious leaders (n = 50), and community leaders (n = 50) from August to December 2019. We used convenience sampling to recruit parents presenting with a child age ≤ 5 years for well or sick care during routine clinic hours at the Trifinio Center for Human Development. For community and religious leaders, a research assistant recruited individuals in-person or over the telephone using previously available contact information obtained through the aforementioned community needs assessment [16]. Eligible community members spoke Spanish and were ≥ 18 years old. Parents who spoke Mayan languages only were ineligible. Eligible religious leaders and community leaders spoke Spanish, were ≥ 18 years old, and were an established religious leaders or community leader in a current role (e.g. politician, teacher, COCODE (Guatemalan Community Council for Urban and Rural Development) member). COCODEs differ from political leaders–i.e., mayors, judges, or magistrates–as they are more locally-relevant community figures without the same political or financial resources available to these others.

### Survey design and administration

We based the survey on the Theory of Planned Behavior, which emphasizes the role of subjective norms–such as those set by community leaders–as they relate to individuals’ preventive healthcare intentions and behaviors (e.g., vaccination) [17]. Surveys included basic demographic questions for all respondents about age, sex, marital status, educational level, income, assets, and family size. Given our intentions to survey religious leaders and other civic leaders and community members, we specifically included questions about religion and religiosity. To assess religion and religiosity, we used the validated Duke University Religiosity (DUREL) tool, which assesses individual religiosity across three domains: religious service attendance, private religious activities (e.g., prayer, meditation), and perceived importance of religious beliefs [18]. The DUREL has been translated into Spanish and multiple other languages [18].

To assess socioeconomic status, we used a modified wealth index based on best practices derived from an 8-country study of wealth in developing countries [19]. Our wealth index was scored on a scale of 0–24, assigning up to 8 points in each of 3 demographic categories (income, assets, education). To assess vaccine hesitancy, we used the 5-question validated Guatemalan Vaccine Attitudes (GuaVA) tool (S1 Table), which is scored from 0 to 10; a score of 0 is considered “not hesitant”, scores of 1–3 are considered “somewhat hesitant”, and scores of 4–10 are considered “hesitant” [4]. We asked community members how strongly they trusted different leaders for advice on childhood vaccines (e.g., A lot, A little, Not Sure). We used simplified Likert scales due to prior vaccination survey work in rural Guatemala that encountered interpretative difficulties with extended Likert scale response options [6]. Additionally, we asked religious leaders and community leaders whether they felt comfortable speaking about childhood vaccines or a perceived responsibility to do so. Finally, we asked leaders about their prior experiences with vaccine advocacy in their communities in the past 12 months. We pre-tested the survey instrument with ten rural Guatemalan public health nurses in an hour-long theatre testing event before pre-testing the survey instrument with an additional ten rural Guatemalan community members and leaders, incorporating their feedback. The final survey instruments for community leaders, religious leaders, and community members are available as supplemental files (S1–S3 Files).

A research assistant and community health worker (NR) approached parents in person at the Southwest Trifinio Center for Human Development or recruited community leaders and religious leaders identified in a prior community needs assessment by phone to schedule a time to discuss the study. Religious leaders and community leaders were contacted up to a maximum of three times by phone. As many community members have limited schooling and/or cannot read, surveys were administered verbally, in person, to all participants. Responses were recorded on paper surveys and uploaded to REDCap (Research Electronic Data Capture), a secure application that permits data capture and storage for research studies [20]. All participants provided written consent prior to participation. There were no incentives. The study was approved by the Colorado Multiple Institutional Review Board (Aurora, CO, USA), approved by the Institutional Review Board of University Francisco Marroquin (Guatemala City, Guatemala), and agreed to by the Community Advisory Board for Research of the Southwest Trifinio area.

### Primary outcome variables, independent variables, and analyses

Our primary outcome variables, independent variables of interest, and analytic approaches differed by study aim. For our first aim, which sought to compare religious leaders’ and community leaders’ attitudes related to childhood vaccines, our primary outcome was GuaVA score, by category noted above [4]. Independent variables included modified wealth index, age, family size, sex, and religion. We calculated descriptive statistics on the primary outcome and independent variables for community members, religious leaders, and community leaders, comparing responses by participant types; we obtained p-values for these comparisons using ANOVA for continuous variables and chi-square or Fisher’s exact tests for categorical variables. We conducted unadjusted proportional odds regression analyses of the association between respondent type and the ordinal outcome of GuaVA score, divided into the three categories described above. We then used multivariable proportional odds regression to measure associations between respondent type and GuaVA score, adjusting for wealth, age, family size, sex, and religious affiliation. The proportional odds regression model we used only requires that the outcome variable be ordinal (not continuous or normally distributed), and so is appropriate for a Likert-type variable.

For our second aim, which sought to describe leaders’ comfort and experiences with vaccine advocacy, our primary outcome variables were ordinal measures of leaders’ comfort levels and perceived responsibility talking about vaccines. We also asked about religious leaders and community leaders’ experiences talking about vaccines in their communities in the prior twelve months. We performed descriptive analyses for these questions, comparing the proportions of community leaders and religious leaders’ categorical responses with Fisher’s exact tests.

Lastly, for our third aim, which sought to describe community members’ levels of trust in various community leaders or religious leaders as vaccine advocates, our primary outcome variable was an ordinal measure of trust. First, we calculated the proportions of community members responding at each trust level for each type of leader; hypothesis testing was conducted in the generalized estimating equations (GEE) framework on the binary outcome of “A lot” of trust versus “Not sure” or “A little” trust, using robust sandwich covariance estimators to account for correlation within respondents [21]. All analyses were performed with R (R Foundation for Statistical Computing, Vienna, Austria).

## Results

We recruited 250 participants (response rate 99.2%; two community members declined). Overall, community members were more likely to be younger, female, poorer, and have fewer children than community leaders or religious leaders (Table 1).

**Table 1:** Participant demographics and Guatemalan Vaccine Attitudes tool (GuaVA) scores, stratified by respondent type. P values are for comparisons across groups^1^.

Variable	Community Members	Community Leaders	Religious Leaders	*P*
	(N = 150)	(N = 50)	(N = 50)	
Sex, N (%)				**< 0.01**
Male	8 (5)	39 (78)	33 (66)	
Female	142 (95)	11 (22)	17 (34)	
Mean Age (SD)	27.3 (6.8)	49.1 (10.8)	50.3 (13.8)	**< 0.01**
Marital Status, N (%)				**0.03**
Single	8 (5)	9 (18)	2 (4)	
Married/Together	137 (91)	37 (74)	46 (92)	
Divorced	1 (1)	2 (4)	1 (2)	
Other	4 (3)	2 (4)	1 (2)	
Number of children,^2^ N (%)				**< 0.01**
1	35 (23)	3 (6)	2 (4)	
2–3	88 (59)	20 (41)	16 (34)	
4–5	18 (12)	15 (31)	13 (28)	
6 or more	9 (6)	11 (22)	16 (34)	
Highest Education Level, N (%)				0.14
None	5 (3)	5 (10)	3 (6)	
Primary school	100 (67)	30 (60)	30 (60)	
Secondary school	28 (19)	5 (10)	6 (12)	
High school	15 (10)	7 (14)	9 (18)	
Advanced degree	2 (13)	3 (6)	2 (4)	
Mean Wealth Index (0–24; higher score indicates wealthier) (SD)	8.8 (3.2)	10.7 (4.4)	10.5 (4.2)	**< 0.01**
Community of Residence, N (%)				0.99
A	48 (32)	18 (36)	16 (32)	
B	84 (56)	27 (54)	28 (56)	
Other	18 (12)	5 (10)	6 (12)	
Religious Affiliation, N (%)				0.12
None	19 (13)	7 (14)	0 (0)	
Catholicism	38 (25)	16 (32)	15 (30)	
Evangelicalism	87 (58)	26 (52)	35 (70)	
Mormonism	2 (1)	0 (0)	0 (0)	
Jehovah’s Witnesses	2 (1)	1 (2)	0 (0)	
Other	2 (1)	0 (0)	0 (0)	
Religious Service Attendance, N (%)				**< 0.01**
Never	14 (9)	3 (6)	0 (0)	
Occasionally	83 (55)	28 (56)	4 (8)	
Weekly or more often	53 (35)	19 (38)	46 (92)	
Private Religious Activities, N (%)				**< 0.01**
Rarely/Never	23 (15)	9 (18)	0 (0)	
Occasionally	102 (68)	32 (64)	21 (42)	
Daily or more often	25 (17)	9 (18)	29 (58)	
Intrinsic Religiosity Score^3^, N (%)				**< 0.01**
3–9 (Least Religious)	1 (1)	1 (2)	0 (0)	
10–14 (Moderately Religious)	47 (31)	10 (20)	1 (2)	
15 (Most Religious)	102 (68)	39 (78)	49 (98)	
GuaVA Score (Interpretation), N (%)				0.71
0 (Not Vaccine Hesitant)	67 (45)	22 (44)	19 (38)	
1–3 (Somewhat Vaccine Hesitant)	61 (41)	22 (44)	24 (48)	
4–10 (Vaccine Hesitant)	22 (15)	6 (12)	7 (14)	

^**1**^ Comparisons with Fisher exact or Chi-Square tests for categorical variables (Fisher exact tests if cell count < 5) and Analysis of Variance (ANOVA) or t-tests for continuous variables.

^2^ There were missing data for one community leader and three religious leaders; proportions are presented for respective denominators of 49 (community leaders) and 47 (religious leaders).

^**3**^ This score is a subscale created by summing participant responses to three separate questions of agreement from the Duke University Religiosity Index^8^ (1. In my life, I experience the presence of the Divine; 2. My religious beliefs are what really lie behind my whole approach to life; 3. I try hard to carry my religion over into all other dealings in life).

Of the 50 participating community leaders, 30 (60%) were COCODEs, 3 (6%) were teachers, 13 (26%) were political leaders, and 4 (8%) held other leadership roles. Most community members, community leaders, and religious leaders identified with Evangelical or Catholic faith traditions, without significant differences in religious affiliation between religious leaders, community leaders, and community members (P = 0.12). Religious leaders were more likely to report attending religious services, engaging in private religious activities, and having very high intrinsic religiosity, compared to community leaders and community members (P < 0.01 for each comparison). One in three community leaders and community members attended religious services weekly or more often, one in six prayed daily or more often, and two-thirds had high intrinsic religiosity.

In bivariable analyses, community members, community leaders, and religious leaders had similar GuaVA scores (P = 0.71); 1 in 7 (14%) of religious leaders or community leaders scored as vaccine hesitant. Table 2 compares religious leaders and community leaders’ responses on individual GuaVA questions.

**Table 2:** Community and religious leaders’ responses to Guatemalan Vaccine Attitudes (GuaVA) items^1^.

Question	Community Leaders, N = 50 (%)			Religious Leaders, N = 50 (%)			*P*
	No	Not Sure	Yes	No	Not Sure	Yes	
Children get more shots than are good for them.	30 (60)	7 (14)	13 (26)	25 (50)	7 (14)	18 (36)	0.53
It is better for my child to develop immunity by getting sick than to get a shot.	44 (88)	2 (4)	4 (8)	46 (92)	1 (2)	3 (6)	0.78
It is better for children to get fewer vaccines at the same time.	40 (80)	3 (6)	7 (14)	38 (76)	2 (4)	10 (20)	0.74
I trust the information I receive about shots.	47 (94)	2 (4)	1 (2)	46 (92)	2 (4)	2 (4)	1.00
	Not Hesitant	Not Sure	Hesitant	Not Hesitant	Not Sure	Hesitant	***P***
Overall, how hesitant about childhood shots would you consider yourself to be?	43 (86)	5 (10)	2 (4)	46 (92)	2 (4)	2 (4)	0.50

^**1**^ Comparisons with Fisher exact or Chi-Square tests (Fisher exact tests if cell count < 5).

Religious leaders and community leaders had similar score distributions across all 5 GuaVA questions. In multivariable models adjusted for age, sex, wealth, religion, and family size, religious leaders and community leaders were still no more likely to be mildly or highly vaccine hesitant, referent to community members (cumulative adjusted ORs 0.86 for community leaders and 1.14 for religious leaders, referent to community members; P > 0.7 for each comparison).

**Fig 1:**
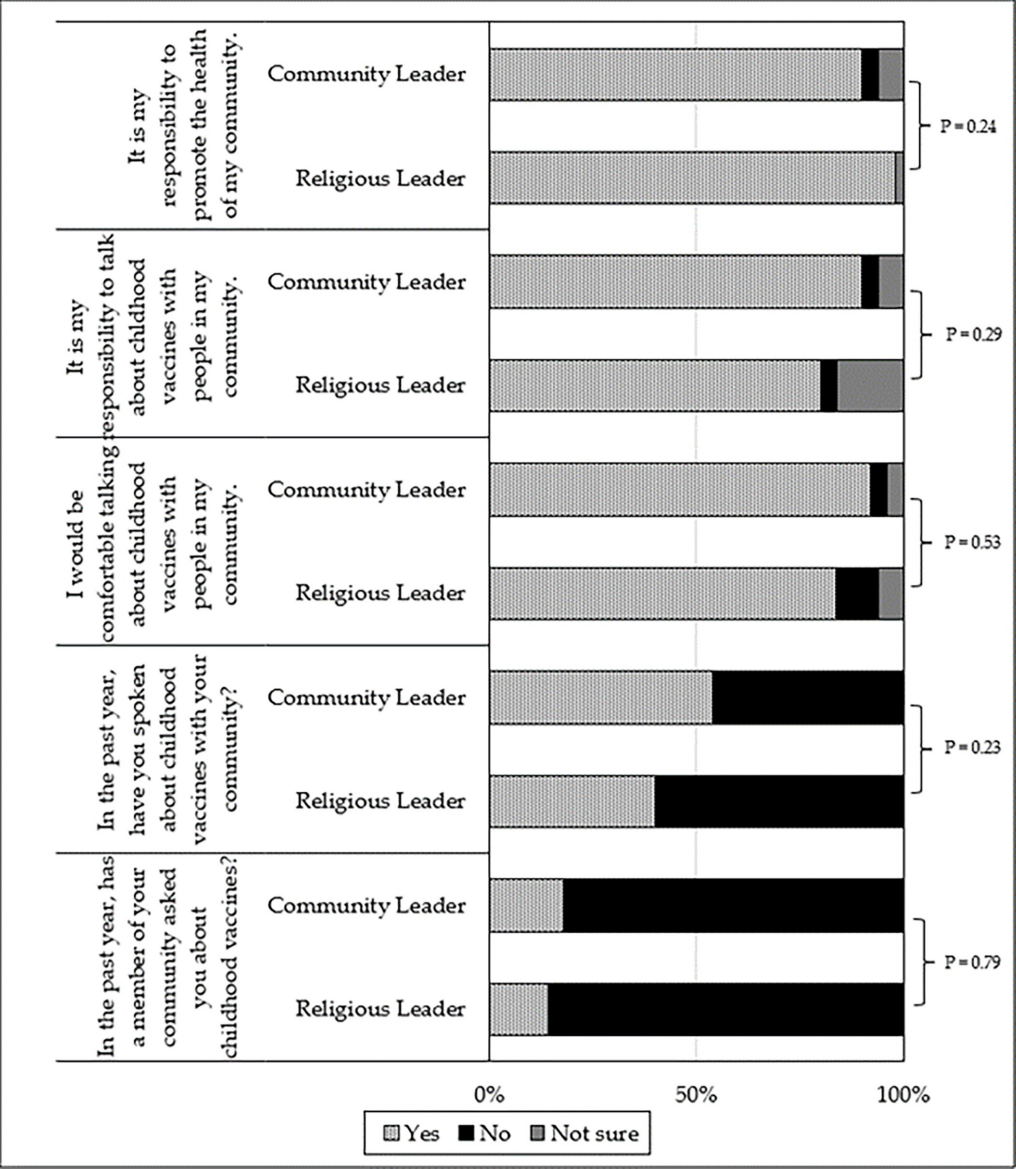
Religious leaders’ and community leaders’ (N = 100) perceived responsibility, comfort levels, and reported experiences with vaccine advocacy. Comparisons between responses performed with Fisher exact tests (as cell counts < 5).

Fig 1 presents religious leaders’ and community leaders’ responses to questions about their perceived responsibilities to promote communal health and vaccination, their comfort talking about vaccines with their community members, and their prior experiences hearing from and speaking to their community members about vaccines.

**Fig 2:**
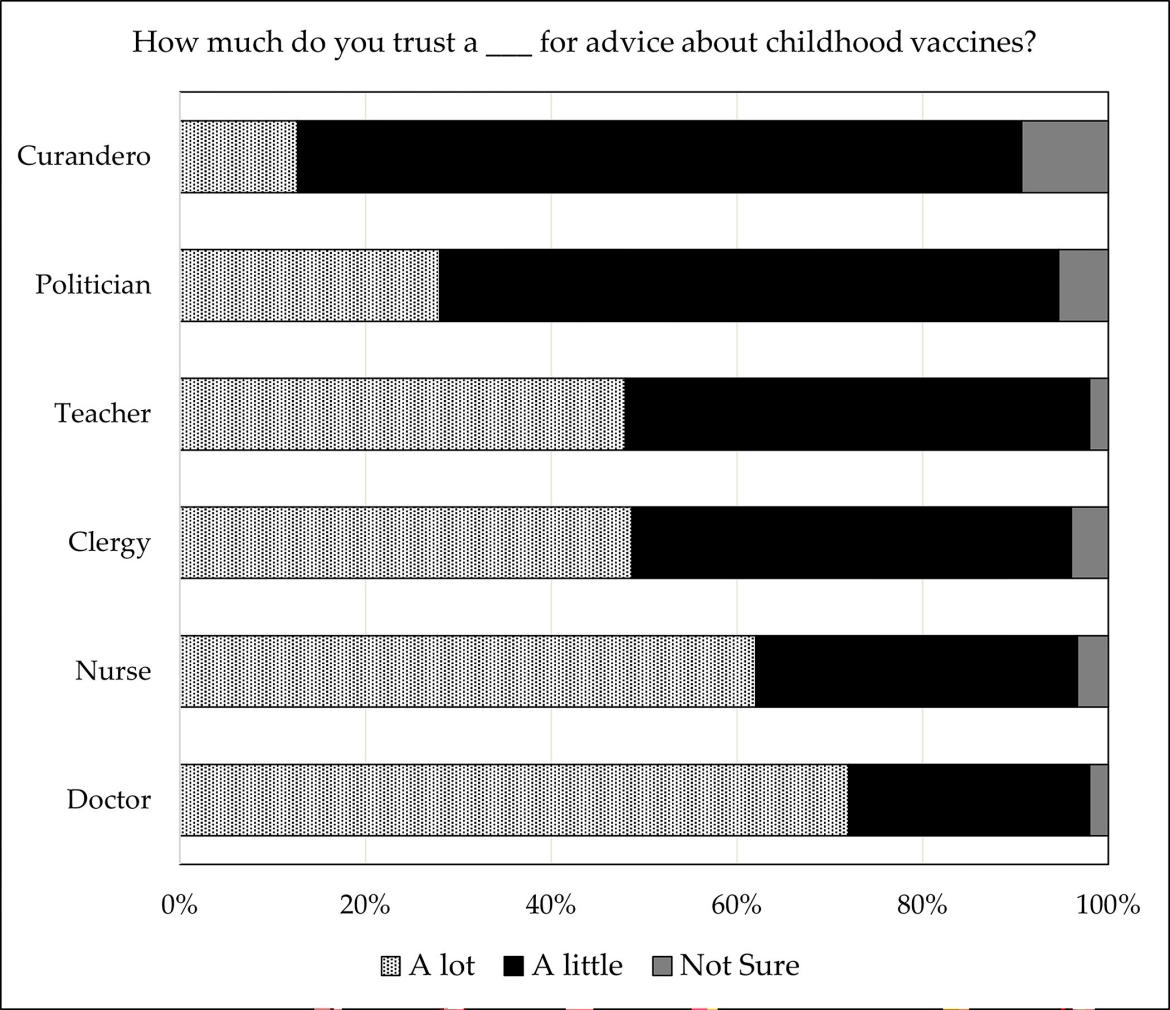
Parental (N = 150) trust in specific leaders for advice about childhood vaccines.

Religious leaders, on average, reported serving in their position for longer than community leaders (13.5 ± 10.9 years vs. 4.4 ± 6.7 years; P < 0.01). Fewer than half of all community and religious leaders had spoken about vaccines to their communities in the prior year, and only one in six leaders reported that a member of their community had asked them about vaccines in the past 12 months. There were no significant differences between leaders on any items (Fig 1). Fig 2 presents community members’ responses to questions about the extent to which they would trust certain leaders for vaccine advice (versus doctors, nurses, and *curanderos*—traditional native healers).

Overall, community members were less likely to report trusting politicians “a lot” for vaccination advice (28%) relative to religious leaders (49%; P < 0.01), teachers (48%; P < 0.01), nurses (62%; P < 0.01), and doctors (72%; P < 0.01). *Curanderos* were least trusted of all leader types.

## Discussion

In this pilot study, few religious leaders or community leaders were vaccine hesitant, most leaders felt responsible to advocate for childhood vaccines, and nurses and doctors were most trusted by community members for vaccination advice. Yet, as nearly half of community members trusted teachers and religious leaders a lot for vaccination advice, our preliminary data encourage future work to mobilize and educate religious leaders and teachers in rural Guatemalan contexts to promote childhood vaccination.

Few religious leaders or community leaders had GuaVA scores considered hesitant, which is encouraging for global public health workers and organizations who have called for more engagement in vaccination advocacy [22, 23]. Nevertheless, one in three leaders expressed concerns that children get more vaccines than are good for them and one in five thought it best for children to get fewer vaccines simultaneously. These concerns mirror those identified among parents in other studies of vaccine hesitancy in Guatemala and comparable developing countries [5, 6]. To address these concerns and others, physicians, nurses, and community health workers involved in local vaccination programs should solicit leaders’ questions and correct mistruths or misperceptions as they hear them.

While nearly all religious and community leaders we sampled felt comfortable and responsible to talk about childhood vaccines in their communities, only half reported advocating in their communities in the prior year. This disconnect between perceived responsibility and reported actions suggests leaders may need concrete cues to action from public health leaders. Qualitative interviews with clergy in American faith communities have found that leaders’ perceptions of congregational health or public health needs influence their perceived responsibility to advocate for vaccines [24]. The COVID-19 pandemic has likely provided many leaders an opportunity to practice advocacy and promote child public health in their places of worship, schools, or jurisdictions. Unfortunately, COVID-19 has posed critical and ongoing threats—such as disrupted well child care, supply chain problems, and staffing shortages—to preventive care and routine immunization services for children [25], especially in low and middle income countries [26]. We suggest public health workers cue community leaders and religious leaders to action to champion routine vaccines as part of catch-up preventive care services during the ongoing pandemic and beyond.

When considering community partners, our data suggest public health workers have many options for vaccination advocacy. Foremost, parents of young children trusted nurses and doctors for vaccination advice. However, one in four respondents in our survey only trusted doctors “a little”. These findings complement those from prior surveys of parents of young children in Guatemala, which found that most parents reported doing what their doctor recommended regarding childhood vaccination [5, 6]. However, in the multi-country study from 2016–2018 in which Guatemala was included, one in five parents reported strongly disagreeing with their healthcare provider’s recommendation on vaccines [5]. For such parents, advocacy from religious leaders or other community leaders may be especially influential. Our study gives public health workers direction as they seek to choose community partners wisely to maximize a return of vaccination confidence on their partnership efforts.

In this survey, most community leaders were COCODEs, or members of Guatemalan Community Councils for Urban and Rural Development. COCODEs are elected by community assemblies and provide representation for communities on local matters. A multiyear study examining the role of these influential leaders in Palencia, Guatemala noted how they have strong community legitimacy and excellent knowledge of local culture, which are fundamental to the implementation of community health programs [27]. The author suggested COCODEs are equally important–if not more important–than political leaders who have financial and political resources necessary to create and fund programs [27]. In our study, the vast majority of community leaders we surveyed–mostly COCODEs–felt comfortable advocating for childhood vaccines, but only half had done so in the prior year. In lieu of seeking advocacy from high-level politicians, vaccination advocates should cue COCODEs to action (and equivalent leaders in comparable developing countries) and engage them in vaccine advocacy. Future work should explore how best COCODES can engage communities to promote childhood vaccines.

In addition to COCODEs, teachers may be especially important vaccination advocates. While teachers comprised a small number of our survey sample, they have been identified as influential child health advocates for a variety of topics in Guatemala, including substance abuse [28], chronic disease prevention [29], suicide prevention [30], and food insecurity [31]. Teachers’ daily interaction with children and families, their involvement in school-based programming, and their longitudinal presence in communities may endear them to parents. Our data suggests vaccination is an additional relevant area for public health engagement, as community members identified teachers as a specific kind of community leader they trusted–as much as religious leaders–for vaccine information. Health officials could collaborate with teachers to host yearly “Back to School” educational events or prior to seasonal influenza circulation. Vaccination requirements for school attendance could be unethically burdensome in rural areas, but teachers could clarify which vaccines children need by grade level and coordinate with parents to review students’ immunization cards annually. Given the low number of teachers in our sample, more work is needed to capture their perspectives.

Finally, public health officials should enlist the help of religious leaders. Our sample of community leaders and community members attended religious services occasionally or more often and had similar overall levels of religious affiliation as found throughout Guatemala [32]. Religious leaders dialogue with community members frequently, and our study suggests they are most trusted after doctors and nurses for child vaccination advice. Given their willingness to help, religious leaders could be recruited as full partners in vaccination advocacy in rural Guatemala and similar contexts. Indeed, many may already regularly organize local public health events. A qualitative study of pediatric chronic disease prevention among 87 Guatemalan parents, teachers, religious leaders, and educational officials found religious leaders routinely lead health-related seminars and workshops for families [29]. Yet, they desired training to better promote healthy lifestyles in their congregations [29]. Public health officials can capitalize on these desires by seeking out religious leaders as partners and offering seminars on key topics: e.g., routine vaccination schedules, principles of individual or herd immunity, and common vaccine concerns and myths. Religious leaders may even desire to set up regular educational events or coordinate vaccine drives.

This sentinel pilot study had several limitations. It occurred in a single rural geographic area, employed a convenience sample design, and had a small number of respondents (n = 250). Furthermore, most community members were young female parents, and it is unclear whether male community members’ attitudes differ toward religious leaders or teachers as vaccination advocates. Recall or social desirability biases may have affected how participants responded to survey questions. Specifically, religious leaders and community leaders in close-knit rural communities may have felt more compelled to participate. Or, those who are already vaccination advocates (or are more inclined to become vaccination advocates) may have been more likely to respond to recruitment efforts and complete surveys. Thus, future work should determine the extent to which our findings are reproducible in urban communities as well as with larger number of community leaders and religious leaders, specifically looking for differences by community leader type. Additional studies may also show whether COVID-19 has changed individuals’ attitudes toward vaccines (including COVID-19 vaccines), community members’ perceptions of leaders as vaccine advocates, and leaders’ perceptions of and experiences with advocacy.

## Conclusions

This sentinel pilot study in rural Guatemala found religious leaders and community leaders were infrequently hesitant about childhood vaccines and were willing vaccination advocates. Half of community members highly trusted teachers and religious leaders for vaccination advice. Public health officials in rural Guatemala should enlist COCODEs, teachers, and religious leaders, address any concerns they have about childhood vaccines, and engage them as complementary partners in the important work of child vaccine advocacy.

## Supporting information

S1 Dataset Deidentified dataset used for study analyses. (CSV)

S1 File Final survey instrument for community leaders. (DOCX)

S2 File Final survey instrument for religious leaders. (DOCX)

S3 File Final survey instrument for community members. (DOCX)

S1 Table Guatemalan Vaccine Attitudes (GuaVA) 5-question tool with questions, responses, and scoring instructions. (XLSX)
