# Supplementary material for: Community perceptions of vaccine advocacy for children under five in rural Guatemala
Source: PLOS Glob Public Health. 2023 May 22;3(5):e0000728. doi: 10.1371/journal.pgph.0000728 (PMC10202271; doi:10.1371/journal.pgph.0000728)
Supplement: S3 File — (DOCX) [file pgph.0000728.s004.docx]

**M€- Date: ________/________/________**

(Community) (Number)

**SURVEY OF COMMUNITY MEMBERS ABOUT VACCINE CONFIDENCE IN THE TRIFINIO AREA OF SOUTHWEST GUATEMALA**

*Thank you for agreeing to participate in this survey. We will ask you questions about your religious beliefs and whether or not your beliefs affect your thoughts about vaccines. There are no good or bad answers. If you are not sure about a questions, you can ask to clarify. We will not share your responses with anyone. If you have a question or concern about this study, you can contact Dr. Guillermo Antonio Bolaños or Dr. Edwin Asturias at one of the telephone numbers that have been provided in the consent form that you have signed.*

| 1 | What municipality do you live in? | 🞏 Coatepeque  🞏 La Blanca  🞏 Caballo Blanco  🞏 Other: _______________________ |
| --- | --- | --- |
| 2 | What town (village) or community do you live in? | Write the name here:  _______________________________ |
| 3 | How old are you? | __ __ years |
| 4 | What is your gender? | 🞏 Male  🞏 Female |
| 5 | What is your marital status? | 🞏 Single  🞏 United (civil union) or married  🞏 Divorced or widowed  🞏 Other: _______________________ |
| 6 | What is your highest educational level? | 🞏 None  🞏 Primary school (1-6 grade)  🞏 Middle/high school (7-9 grade)  🞏 Technical training  🞏 University  🞏 Other: _______________________ |
| 7 | Approximately, how much money do you receive monthly? | 🞏 No income  🞏 < 1,000 Quetzales  🞏 Q. 1,000-3,000  🞏 Q. 3,001-6,000  🞏 Q. 6,001-10,000  🞏 Q. Mas de 10,000 |
| 8 | Do you have any of the following items in your home?  *Please mark all of the following.*  Radio  Television  Cell phone  Computer  Internet  Refrigerator  Gas stove  Electric stove | 🞏 Yes 🞏 No  🞏 Yes 🞏 No  🞏 Yes 🞏 No  🞏 Yes 🞏 No  🞏 Yes 🞏 No  🞏 Yes 🞏 No  🞏 Yes 🞏 No  🞏 Yes 🞏 No |

| 9 | Do you have any of the following vehicles in your house?  *Please mark all of the following.*  Bicycle  Motorcycle  Car or pickup truck  Van (minibus)  Boat | 🞏 Yes 🞏 No  🞏 Yes 🞏 No  🞏 Yes 🞏 No  🞏 Yes 🞏 No  🞏 Yes 🞏 No |
| --- | --- | --- |
| 10 | How many living children do you have?  *If the answer is (0), skip to question 12* | Number of children: ___ |
| 11 | List all of your children, their genders and their ages  First  Second  Third  Fourth  Fifth  Sixth  Seventh | 🞏 M 🞏 F Age:___ ___ years  🞏 M 🞏 F Age:___ ___ years  🞏 M 🞏 F Age:___ ___ years  🞏 M 🞏 F Age:___ ___ years  🞏 M 🞏 F Age:___ ___ years  🞏 M 🞏 F Age:___ ___ years  🞏 M 🞏 F Age:___ ___ years |
| 12 | What religion were you baptized into? | 🞏 I was not baptized  🞏 Catholic  🞏 Evangelical  🞏 Mormon  🞏 Jehovah’s Witness  🞏 Maya religion  🞏 Other: _______________________  🞏 I don’t know |
| 13 | What religion do you currently belong to? | 🞏 None  🞏 Catholic  🞏 Evangelical  🞏 Mormon  🞏 Jehovah’s Witness  🞏 Maya religion  🞏 Other: _______________________ |
|  | *For the following statements, mark the best response* | |
| 14  (D1) | How often do you go to church, other religious services, or other religious gatherings? *(at least)* | 🞏 Never  🞏 Once a year or less  🞏 A few times per year  🞏 A few times per month  🞏 Once a week  🞏 More than once a week |
| 15  (D2) | How often do you dedicate your time to private religious activities such as praying, meditating, or studying the Bible? | 🞏 Rarely or never  🞏 Once a month or less  🞏 Once a week  🞏 A few times a week  🞏 Once a day  🞏 More than once a day |

|  | *For the following statements, mark if it is true or not true for you.* | |
| --- | --- | --- |
| 16  (D3) | In my life, I feel a divine presence *(for example, of God).* | 🞏 Definitely not true  🞏 Sometimes not true  🞏 Neutral  🞏 Sometimes true  🞏 Definitely true |
| 17  (D4) | My religious beliefs guide or define what I do in my life. | 🞏 Definitely not true  🞏 Sometimes not true  🞏 Neutral  🞏 Sometimes true  🞏 Definitely true |
| 18  (D5) | I make an effort to apply my religion in all aspects of my life. | 🞏 Definitely not true  🞏 Sometimes not true  🞏 Neutral  🞏 Sometimes true  🞏 Definitely true |
|  | *For the following, mark the best response* | |
| 19 | My religion agrees with vaccines | 🞏 Yes  🞏 No  🞏 I’m not sure  🞏 I’m not religious |
| 20 | How much do you trust the advice of the following people when you are making decisions about your health?  *Please mark each of the options.*  Doctor  Nurse  Midwife  Traditional healer  Family  Close friends  Political or community leaders  Religious leaders  Teachers | 🞏 A lot 🞏 A little 🞏 I am not sure  🞏 A lot 🞏 A little 🞏 I am not sure  🞏 A lot 🞏 A little 🞏 I am not sure  🞏 A lot 🞏 A little 🞏 I am not sure  🞏 A lot 🞏 A little 🞏 I am not sure  🞏 A lot 🞏 A little 🞏 I am not sure  🞏 A lot 🞏 A little 🞏 I am not sure  🞏 A lot 🞏 A little 🞏 I am not sure  🞏 A lot 🞏 A little 🞏 I am not sure |
| *For the following questions, mark if you agree (yes), do not agree (no), or are not sure.* | | |
| 21  (P4)* | Do you believe that children receive more vaccines than are good for them? | 🞏 Yes 🞏 No 🞏 I am not sure |
| 22  (P5) | Do you believe that many of the illnesses that vaccines prevent are serious? | 🞏 Yes 🞏 No 🞏 I am not sure |
| 23  (P6)* | Do you believe that its better for children to develop immunity naturally rather than through a vaccine? | 🞏 Yes 🞏 No 🞏 I am not sure |
| 24  (P7)* | Do you believe that it would be better if children received fewer vaccines? | 🞏 Yes 🞏 No 🞏 I am not sure |

| *For the following questions, mark the best answer* | | |
| --- | --- | --- |
| 25  (P9) | How worried are you that one of the childhood vaccines may not be safe? | 🞏 Very 🞏 A little 🞏 I am not sure |
| 26  (P10) | How worried are you that vaccines may not prevent disease? | 🞏 Very 🞏 A little 🞏 I am not sure |
| 27  (P12)* | In general, how insecure do you feel with respect to childhood vaccines? | 🞏 Not at all insecure  🞏 I am very insecure  🞏 I don’t know |
| 28  (P13)* | Do you trust the information that you receive about vaccines? | 🞏 Yes 🞏 No 🞏 I am not sure |
| 29  (WL1) | Do you believe that vaccines are important for the health of children? | 🞏 Yes 🞏 No 🞏 I am not sure |
| 30  (WL3) | Do you believe that vaccinating children is important to protect the health of the rest of the people in my community? | 🞏 Yes 🞏 No 🞏 I am not sure |
| 31  (WL7) | Do you believe that getting vaccines is a good way to prevent disease? | 🞏 Yes 🞏 No 🞏 I am not sure |
| 32  (WQ8) | Do you believe that it’s difficult for some religious groups in the community to obtain vaccines?  *If your answer is yes, which groups?*  *If your answer is yes, why is it difficult?* | 🞏 Yes 🞏 No 🞏 I am not sure  _______________________________  _______________________________  _______________________________  _______________________________  _______________________________  _______________________________ |
| 33  (WQ9) | Have you received or heard negative information about vaccination?  *If your answer is yes, what type of negative information?*  *If your answer is yes, who did you receive this information from?* | 🞏 Yes 🞏 No 🞏 I am not sure  _______________________________  _______________________________  _______________________________  _______________________________  _______________________________  _______________________________ |
| 34  (WQ10a) | Religious leaders in my community support childhood vaccines | 🞏 Yes 🞏 No 🞏 I am not sure |
| 35  (WQ10b) | Political leaders in my community support childhood vaccines | 🞏 Yes 🞏 No 🞏 I am not sure |
| 36 (WQ10c) | Teachers In my community support childhood vaccines | 🞏 Yes 🞏 No 🞏 I am not sure |
| ***If you have children, continue with this survey. If you DO NOT have children, this survey is finished!*** | | |

| 37 | How much do you trust your own experiences when making decisions about the health of your child? | 🞏 A lot 🞏 A little 🞏 I’m not sure |
| --- | --- | --- |
| 38 | How much do you trust the advice of the following people when making decisions about vaccinating your child?  *Please mark each of the options.*  Doctor  Nurse  Midwife  Traditional healer  Family  Close friends  Political or community leaders  Religious leaders  Teachers | 🞏 A lot 🞏 A little 🞏 I’m not sure  🞏 A lot 🞏 A little 🞏 I’m not sure  🞏 A lot 🞏 A little 🞏 I’m not sure  🞏 A lot 🞏 A little 🞏 I’m not sure  🞏 A lot 🞏 A little 🞏 I’m not sure  🞏 A lot 🞏 A little 🞏 I’m not sure  🞏 A lot 🞏 A little 🞏 I’m not sure  🞏 A lot 🞏 A little 🞏 I’m not sure  🞏 A lot 🞏 A little 🞏 I’m not sure |
| 39 | In your family, who makes the decision to vaccinate or not vaccinate your children? | 🞏 Father  🞏 Mother  🞏 Both parents  🞏 Other: _______________________ |
| 40  (P1) | Have you ever postponed getting a vaccine for your child for reasons other than illness or allergy? | 🞏 Yes 🞏 No 🞏 I’m not sure |
| 41  (P2) | Have you ever not given your child a vaccine for reasons other than illness or allergy? | 🞏 Yes 🞏 No 🞏 I’m not sure |
| 42  (P3) | How sure are you that giving your child all of the recommended vaccines is good for them? | 🞏 I am not sure  🞏 I am a little sure  🞏 I am very sure |
| 43  (P8) | How worried are you that your child could have side effects to a vaccine? | 🞏 Very 🞏 A little 🞏 I’m not sure |
| 44  (P11) | If you have another baby now, would you want them to receive all of the recommended vaccines? | 🞏 Yes 🞏 No 🞏 I’m not sure |
| 45  (P14) | Can you speak openly with your child’s doctor about your questions regarding vaccines? | 🞏 Yes 🞏 No 🞏 I’m not sure |
| 46  (P15) | Taking everything into consideration, how much do you trust your child’s doctor? | 🞏 Not at all  🞏 A little  🞏 A lot |
| 47  (WQ5) | Which of the following are a problem for your child when it comes to getting vaccines in a clinic or health center?  *Please mark all of the following options.*  There are not vaccines in my community  It is very far from my house  The clinic’s schedule  Wait times at the clinic  The cost of transportation  Other | 🞏 Yes 🞏 No  🞏 Yes 🞏 No  🞏 Yes 🞏 No  🞏 Yes 🞏 No  🞏 Yes 🞏 No  🞏 Explain:  _______________________________  _______________________________  _______________________________ |
| ***If the participant has children under the age of 5, ask for their immunization card and complete this page***  *If they do not have children under the age of 5 or if they don’t have their vaccination card, this survey is finished!* | | |

NEUMOCOCO1

NEUMOCOCO1

NEUMOCOCO1

NEUMOCOCO1

NEUMOCOCO1

NEUMOCOCO2

NEUMOCOCO2

NEUMOCOCO2

NEUMOCOCO2

NEUMOCOCO2

NEUMOCOCO3

NEUMOCOCO3

NEUMOCOCO3

NEUMOCOCO3

NEUMOCOCO3
